# PREPARING FOR THE FUTURE OF TECHNOLOGY TO SUPPORT OLDER ADULTS: PERSPECTIVES FROM THE CREATE CENTER

**DOI:** 10.1093/geroni/igac059.857

**Published:** 2022-12-20

**Authors:** Patricia Heyn

## Abstract

The rapid advancement of technology promises new opportunities to help older adults maintain health, wellbeing, community and productive engagement, and purpose in life. However, the potential of technological innovation will not be met unless technology solutions account for the needs, preferences, and abilities of older users and involve older adults in all stages of the design process. This has been the primary focus of the Center for Research and Education on Aging and Technology Enhancement (CREATE). This symposium will discuss threats to the promise of these solutions and approaches to overcome these barriers. This session will start with N. Charness presenting an overview of digital inequity and the current state of the age-related "digital divide." J. Sharit will then present an experimental study examining older adults' willingness to adopt new technologies and attitudinal barriers to adoption. W. Boot will discuss CREATE research that has focused on the potential of virtual reality to improve the lives of older adults and potential facilitators and barriers to successful virtual reality experiences among older adults. W. Rogers will discuss the potential of voice interfaces for emerging technologies, challenges related to the success of this approach, and research gaps. Finally, S. Czaja will conclude with a broad discussion of future applications of technology to support older adults, including how developments in artificial intelligence, sensing technologies, and robotics that can be used to foster everyday activities, cognitive, physical, and emotional health.

